# Do Honeybees Shape the Bacterial Community Composition in Floral Nectar?

**DOI:** 10.1371/journal.pone.0067556

**Published:** 2013-07-03

**Authors:** Yana Aizenberg-Gershtein, Ido Izhaki, Malka Halpern

**Affiliations:** 1 Department of Evolutionary and Environmental Biology, Faculty of Natural Sciences, University of Haifa, Mount Carmel, Haifa, Israel; 2 Department of Biology and Environment, Faculty of Natural Sciences, University of Haifa, Oranim, Tivon, Israel; Wageningen University, The Netherlands

## Abstract

Floral nectar is considered the most important reward animal-pollinated plants offer to attract pollinators. Here we explore whether honeybees, which act as pollinators, affect the composition of bacterial communities in the nectar. Nectar and honeybees were sampled from two plant species: *Amygdalus communis* and *Citrus paradisi*. To prevent the contact of nectar with pollinators, *C. paradisi* flowers were covered with net bags before blooming (covered flowers). Comparative analysis of bacterial communities in the nectar and on the honeybees was performed by the 454-pyrosequencing technique. No significant differences were found among bacterial communities in honeybees captured on the two different plant species. This resemblance may be due to the presence of dominant bacterial OTUs, closely related to the *Arsenophonus* genus. The bacterial communities of the nectar from the covered and uncovered *C*. *paradisi* flowers differed significantly; the bacterial communities on the honeybees differed significantly from those in the covered flowers’ nectar, but not from those in the uncovered flowers’ nectar. We conclude that the honeybees may introduce bacteria into the nectar and/or may be contaminated by bacteria introduced into the nectar by other sources such as other pollinators and nectar thieves.

## Introduction

High sugar concentration which generates high osmotic pressure [1], [2] and a nectar-related protein [3], [4] have been suggested as limiting factors for microbial growth in floral nectar. Despite these potentially restrictive factors, various microorganisms inhabit floral nectar: filamentous fungi, true yeasts, and yeast-like fungi [5]–[8]. Gilliam et al. [9] examined the floral nectar of three different plant species and found that out of 23 samples of *Citrus* nectar only three contained some gram-negative unidentified bacteria. They were unable to isolate bacteria from cotton (*Gossypium* spp.) and prickly pear cactus (*Opuntia* sp.) nectar flowers.

Recently we demonstrated for the first time that bacterial communities in nectar are abundant and diverse, and display significant variation among three plant species: *Amygdalus communis, Citrus paradisi* and *Nicotiana glauca* [10]. Bacterial counts (by DAPI staining) in the floral nectar of these plants were about 10^4^–10^5^ bacteria per one flower [10]. Following our study, Alvarez-Perez et al. [11] showed that bacteria are common inhabitants of the floral nectar of South African wild plants. Alvarez-Perez and Herrera [12] demonstrated that culturable communities of nectar microorganisms associated with wild Mediterranean plants from Southern Spain, are nonrandom assemblages of bacterial and yeast species.

Although many biologists have embraced microbial model systems as tools to address genetic and physiological questions, the explicit use of microbial communities as model systems in ecology has traditionally been more restricted [13]. Microorganisms represent the “unseen majority” of species, individuals and biomass in many ecosystems, hence may also play key roles in community and ecosystem function [14], [15]. As micro- and macro-organisms seem to follow the same general ecological laws and patterns, microbial model system studies could greatly impact our understanding of the ecology of plants and their pollinators. For example, microbial communities in nectar may affect the nectar’s chemical profile, thus directly controlling nectar consumption by flower visitors such as pollinators and nectar thieves, and thereby indirectly governing plant fitness [2], [8], [16], [17]. Brysch-Herzberg [1] examined yeast communities associated with nectar and flower-visiting insects. He concluded that yeasts may use flowers for mass reproduction and as a platform from which they will meet their next host insect for dispersal.

More than 200 plant species of the European flora are known to be visited by honeybees (*Apis mellifera L.*) [18]. The individual bee, however, tends to stick to one kind of flower over a certain period of time, and ingests nectar of this flower species only [19], [20]. Free [21], for example, found that 94% of all pollen foragers collected one pollen type during a foraging trip. Nectar foragers quickly learn flower attributes such as color, shape and odor, and use this information to land selectively on particular flowers [20].

Microbial communities in honeybee intestines have been studied intensively by cultivation-dependent techniques [22], [23] and molecular methods [24]–[27], yet as far as we know bacterial community composition (BCC) obtained from the honeybee surface and mouthparts has not been described. The bee uses its long proboscis to collect the nectar from floral nectaries. While feeding, the external surface of the bee’s other front organs may come into contact with the nectar. Thus, its surface bacteria, including those on its mouthparts, might play a role in the mutual relation of flower and bee.

The goal of our study was to understand the role of bees in shaping the BCC in floral nectar. Honeybee pollinators may transfer bacteria into and out of the nectar, thus manipulating the floral BCC. To better understand the relation of the BCCs on honeybees and in nectar, we studied two plant species in northern Israel (*Amygdalus communis* and *Citrus paradisi*), which are pollinated by honeybees (*Apis mellifera*), by both cultural and non-cultural methods (454-pyrosequencing). First we compared the BCC in the nectar of uncovered and covered *C. paradisi* flowers; next we compared the BCC on the bees captured on the two different plant species with the BCC of the floral nectar of the plant from which they were captured.

## Methods

### Study Site and Sample Collection

Floral nectar and honeybees were collected from two plant species: *Citrus paradisi* (grapefruit) and *Amygdalus communis* (almond), sampled respectively in January-February and March-April 2010. Sampling ranged across an area 20 km in diameter in open areas in northern Israel (around Oranim College, Tivon). We chose these two plant species because both are pollinated by the same honeybee species (*A. mellifera*). To prevent the contact of nectar with pollinators some flower buds of *C. paradisi* were entirely covered with net bags (45×30 cm nylon fly net) for a few days before blooming (covered flowers). Each plant was sampled on a different day. The nectar was collected from about 100 flowers of each sampled plant (five different *A. communis* plants and three different *C. paradisi* plants). Nectar collection from the covered and uncovered flowers was carried out simultaneously. About 700 µl nectar were collected from flowers of each individual plant with sterile tips under sterile conditions to avoid contamination. Three to four honeybees were captured from each sampled plant while they were visiting the flowers, and were transferred immediately to a sterile 50 ml falcon tube. Sterile saline water (15 ml; 0.85% NaCl), supplemented with 1% Glycerol and 1% Tween 80, was added and the tubes were sonicated for 4 min at 25°C in an ultrasonic cleaning bath (40 kHz; Bransonic 32, MRC, Israel) to dislodge bacteria from the bee surfaces. The resulting suspension was used to culture bacteria and extract DNA (as described below). In some cases, when the bees were transferred to the tube their mouthparts were accidentally pulled off, including the mandibles and the proboscis (tongue), and thus were exposed to the dislodging processes as well (as the bee’s mouthparts were not pulled off deliberately it is unlikely that the gut content contaminated the sample). In sum, five different *A. communis* plants were sampled (nectar was sampled from uncovered flowers and honeybees were captured on each plant) and three different *C. paradisi* plants (nectar was sampled from covered and uncovered flowers and honeybees were captured on each plant). The nectar samples from the uncovered flowers and from their honeybees were cultured and the 454-pyrosequencing technique was executed (details are given below). Nectar samples from covered flowers of *C. paradisi* plants were analyzed only by the 454-pyrosequencing technique.

### DNA Extraction

The bacterial suspension resulting from bee sonication was centrifuged at 8,000 g for 10 min, and re-suspended in 200 µl saline. DNA was extracted from this saline solution (200 µl) and from nectar (100 µl). A DNA isolation kit (DNeasy Blood and Tissue, Qiagene, Germany) was used for DNA extraction, according to the manufacturer’s instructions.

### Isolation and Identification of Culturable Bacteria from Nectar and Bees

Bee samples (after sonication) and nectar samples from uncovered flowers were serially diluted, and 0.1 ml aliquots were spread onto R2A agar (Himedia) and R2A agar supplemented with 20% sucrose. The plates were incubated under aerobic conditions at 30°C for 48 h, and then colony-forming units (cfu) were counted in the nectar samples from the uncovered flowers.

Individual colonies from the agar plates were randomly selected according to different morphologies, and streaked again on the appropriate agar plate from which the colony had been sampled to yield single colonies. The isolated colonies were sub-cultured at least five times before identification. Universal bacterial primers 11F and 1512R were used to amplify internal fragments of 16S rRNA genes, according to Felske et al. [28]. The procedure of the 16S rRNA gene amplification was performed in accordance with Izhaki et al. [29]. The amplified PCR products (approximately 1,500 bp) were sequenced in MCLAB laboratories (California, USA). The sequences length were at least 850 bp or more. For the identification of closest relatives, newly determined sequences were compared with those available in the EZtaxon software, version 2.1 (http://www.eztaxon.org; [30]).

### Nucleotide Sequence Accession Numbers

The sequences from this study (cultured isolates) were deposited in the GenBank database under these accession numbers: JQ638273-JQ638275, JQ638280-JQ638283, JQ638299-JQ638320, JQ638324-JQ638329, JQ638331-JQ638336, JQ638339-JQ638343, JQ638347-JQ638352, JQ638355 and JQ638357-JQ638360.

### 454-pyrosequencing of 16S rRNA Genes

Bacterial diversity in nectar from covered and uncovered flowers (uncovered flowers were sampled only from *C. paradisi*) and on bees was analyzed by means of bacterial tag-encoded FLX 454-pyrosequencing (bTEFAP) [31], [32] of 16S rRNA genes (ten samples for *A. communis* and nine samples for *C. paradisi*). This technique was performed by the Research and Testing Laboratory (Lubbock, TX), based on RTL protocols (www.researchandtesting.com). Amplicon lengths were 200–450 bp from the 27F region numbered in relation to the *E. coli* rRNA gene (GGCGVACGGGTGAGTAA) (see Table S1 for full primers and barcode data). A total of ca. 10,000 sequences per sample was obtained.

### Sequence Analysis

The data derived from the sequencing was processed using MOTHUR program (version 1.17.3) [33]. We used the *trim.seqs* script to remove the forward primer and the barcodes. We removed short sequences (<200 bp), sequences with ambiguous base calls, and sequences with homopolymer runs exceeding 6 bp. The raw data is available at the following website: https://sites.google.com/site/articalrawdata.

Sequences were aligned in MOTHUR against the Ribosomal Database Project (RDP) classifier (http://pyro.cme.msu.edu). We removed the taxon “*Cyanobacteria*” from the FASTA file using the *remove.lineage* script. After omitting chimeras and poor quality sequences, we compared the membership and structure of the 19 communities using an OTU-based approach. We generated a distance matrix (using the *dist.seqs* script which calculates uncorrected pairwise distances between aligned DNA sequences). Next we assigned our sequences to OTUs using the *cluster* script with the average neighbor algorithm. The cutoff level was 0.03.

This distance matrix was clustered into OTUs, compiling the OTUs to the most relevant taxonomic level based on percent identity (>97% species, 90%–97% genus, 85%–90% family, 80%–85% order, 77–80% phylum,<77% unclassified). Principal Coordinate Analysis (PCoA) and AMOVA analyses were performed with this distance matrix.

Rarefaction curves (as a measure of diversity) describing the number of OTUs observed as a function of sampling effort were calculated at 3% sequence divergence (using *rarefaction.single* script). We used the *summary.single* script to calculate the sample coverage and richness estimators. Richness is expressed as the number of observed OTUs. Richness was also estimated by the abundance-based coverage estimator (ACE), which is a nonparametric richness estimator based on distribution of abundant (>10) and rare (≤10) OTUs, and the richness estimator Chao1, which is a nonparametric richness estimator based on distribution of singletons and doubletons.

## Results

### Culturable Bacterial Community Composition in Nectar and on Bees

Bacterial colony-forming units (cfu) per ml flower nectar that were collected from two plant species *A. communis* and *C. paradisi* were 1.5 ×10^7^ (±1.3×10^7^) and 9.2 ×10^6^ (±6.9×10^6^) respectively. Representative bacterial isolates were identified from both nectar and body surface, including the mouthparts of the honeybees captured from the two studied plant species (Table 1). The bacterial colonies were selected for the purpose of locating the variety of different culturable bacterial species; therefore colonies of different appearance were chosen and identified. Bacterial species belonging to the *Gammaproteobacteria, Actinobacteria* and *Bacilli* classes were identified in the floral nectar. Species from these bacterial classes, and also from *Alphaproteobacteria*, were identified on bee surfaces and mouthparts (Table 1). One of the most abundant bacterial species in almost all the samples (except bees from *C. paradisi*) was a novel, unidentified *Enterobateriaceae* species, closely related to *Phaseolibacter flectens* [34] (Table 1).

**Table 1:** List of bacterial isolates from nectar and bees of *Amygdalus communis* and *Citrus paradisi*.

		*Citrus paradisi*		*Amygdalus communis*	
Class	Closest relative in GenBank database	Nectar	Bees	Bees	Nectar
*Alpha-proteobacteria*	*Methylobacterium rhodesianum*			1 (99.8)	
	*Methylobacterium populi*			1 (100)	
*Gamma-proteobacteria*	***Acinetobacter lwoffii***				1 (95.9)
	***Erwinia amylovora***			**1 (96.8)**	
	*Klebsiella oxytoca*		1 (98.1)		
	*Pantoea eucrina*	1 (98.6)			
	*Pantoea septica*	2(98.7–99.1)			
	***Phaseolibacter flectens***	**2 (96.6–96.7)**		**5 (95.6–96.6)**	**7 (95.9–96.8)**
*Actinobacteria*	*Microbacterium foliorum*	3 (98.9–99.1)			
	*Micrococcus yunnanensis*			2 (99.8–99.9)	
*Bacilli*	*Bacillus aryabhattai*			1 (100)	
	*Bacillus cereus*		3 (99.5–100)		
	*Bacillus megaterium*			1 (99.7)	
	***Bacillus niabensis***			**1 (96.8)**	1 (98.6)
	*Bacillus oceanisediminis*		1 (99.3)		
	*Bacillus safensis*		1 (100)		1 (100)
	*Bacillus tequilensis*			1 (99.8)	
	*Lactobacillus kunkeei*		3 (100)	6 (100)	
	*Rummeliibacillus stabekisii*		1 (99.7)		
	*Staphylococcus epidermidis*			2 (100)	1 (100)
	*Staphylococcus haemolyticus*			1 (100)	1 (100)
	*Staphylococcus pasteuri*		3 (99.8–99.9)		
	*Terribacillus goriensis*	1 (100)			
	*Terribacillus saccharophilus*	1 (99.3)			

The number before the parentheses indicates the number of isolates, the number within the parentheses indicates the percentage of the 16S rRNA gene similarities to the closest known species. Isolates with less than 97.5% 16S rRNA gene similarities to known species are most likely novel species and the name of their closest relative species is marked in bold. The isolates were identified by comparing their 16S rRNA gene sequences to that of the GenBank database (EZtaxon software version 2.0.http://147.47.212.35∶8080). The sequences were deposited in the GenBank database under the following accession numbers: JQ638273-JQ638275, JQ638280-JQ638283, JQ638299-JQ638320, JQ638324-JQ638329, JQ638331-JQ638336, JQ638339-JQ638343, JQ638347-JQ638352, JQ638355 and JQ638357-JQ638360.

### 454-pyrosequencing Analysis of Bacterial Community Composition in Nectar and on Bees

The 454-pyrosequencing analysis was used to characterize bacterial communities in nectar of uncovered flowers, nectar of covered flowers, and honeybee surfaces and mouthparts collected from three *C. paradisi* plants (nine samples in all, for *C. paradisi*). Bacterial communities were also characterized in the nectar of uncovered flowers and honeybee surfaces and mouthparts collected from five *A. communis* plants (ten samples in all for *A. communis*).

**Figure 1:**
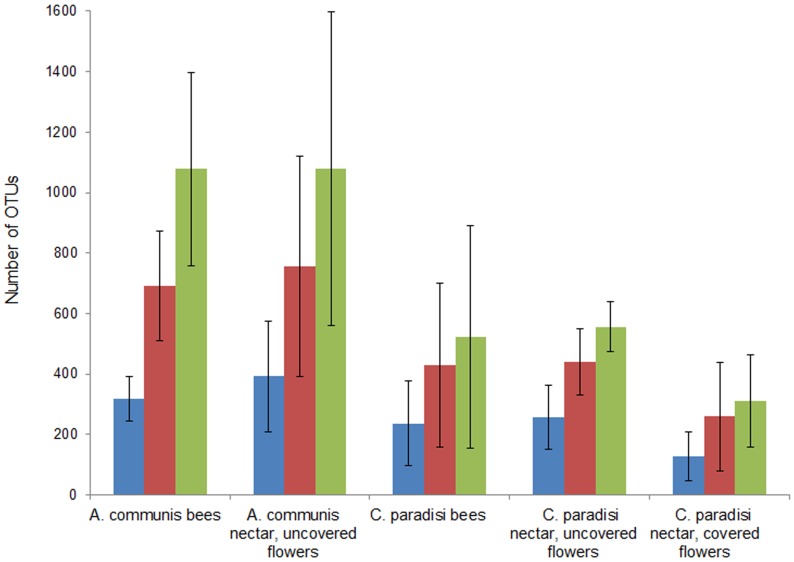
Bacterial richness estimates of *Amygdalus communis* and *Citrus paradisi* representing different management types at a genetic distance of 3%. Richness is expressed as the number of observed OTUs. In addition, richness was estimated by the abundance-based coverage estimator (ACE), which is a nonparametric richness estimator based on distribution of abundant (>10) and rare (≤10) OTUs, and the richness estimator Chao1, which is a nonparametric richness estimator based on distribution of singletons and doubletons. Richness prediction from ACE is colored green, richness prediction from Chao1 red, and richness observed blue.

Sequences were assigned to species-level operational taxonomic units (OTUs) by means of a 97% pairwise-identity cutoff. Across all 19 samples we obtained 78,826 quality sequences. These were classified for a total of 3,330 unique bacterial operational taxonomic units (OTUs) at the 97% sequence-similarity level across all samples, with an average of 277 OTUs per sample. Rarefaction curves at 3% sequence divergence were calculated (Figs. S1 and S2). Some rarefaction curves did not saturate, indicating that the surveying effort did not cover the full extent of taxonomic diversity at this genetic distance. Coverage, Chao1 and ACE richness estimator are shown in Table S2. Bacterial species richness in the nectar and on bees from *C. paradisi* was much lower compared with those from *A. communis* (Table S2, Fig. 1). Full OTU data with their taxonomic classifications and their abundance within each sample are shown in Table S3.

**Figure 2:**
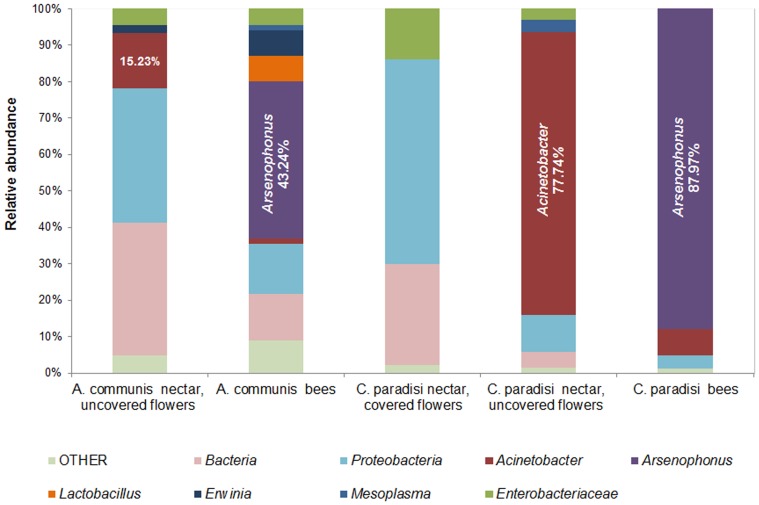
Relative abundances of bacterial taxonomic groups of all sequences derived from the *Amygdalus communis* and *Citrus paradisi* nectar samples and from honeybees captured on the two different plants. Bacterial diversities of all nectar and bee samples were surveyed by 454-pyrosequencing of 16S rRNA genes. Full OTU data with their taxonomic classifications and their abundance within each sample is available in Table S3.

Most sequences from the nectar and bee samples were classified as *Gammaproteobacteria* (76% in average). The most dominant genus in the bee samples was *Arsenophonus*, with a frequency of 43% in bees collected from *A. communis* and of 88% in bees collected from *C. paradisi* (Fig. 2). *Acinetobacter* was the prevalent genus in the nectar of uncovered flowers extracted from both tree species, and it dominated the nectar of uncovered flowers of *C*. *paradisi*, with a frequency of 78% (its frequency in *A. communis* was 15%). The relative abundance of the taxonomic groups is shown in Fig. 2.

**Figure 3:**
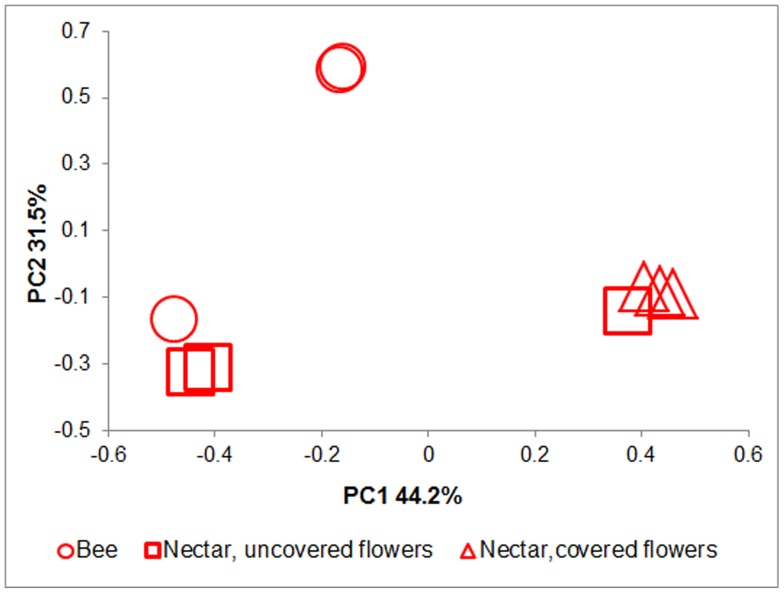
*Citrus paradisi* bacterial diversity clustering by sample source. No significant differences were found in the BCC between honeybees and nectar from uncovered flowers (F_1,4_ = 1.92 P = 0.08). The BCC of covered flowers was significantly different from that of uncovered flowers (F_1,4_ = 2.69; P<0.01) and honeybees (F_1,4_ = 4.36; P<0.01). Bacterial diversities of all samples were surveyed by 454-pyrosequencing of 16S rRNA genes. The first two principal coordinates (PC1 and PC2) from the principal coordinate analysis of weighted UniFrac are plotted for each sample.

Samples of nectar from covered and uncovered flowers and bees captured from *C*. *paradisi* plants were analyzed separately (nine samples in all) and revealed that the BCC found in the nectar of uncovered flowers was similar to that found on the honeybees; the BCC from the covered flowers was significantly different from both BCCs described above (Fig. 3) (AMOVA between the nectar of uncovered and covered flowers F_1,4_ = 2.69; P<0.01; between covered flowers and bees F_1,4_ = 4.36; P<0.01; between bees and the nectar of uncovered flowers F_1,4_ = 1.92; P = 0.08).

**Figure 4:**
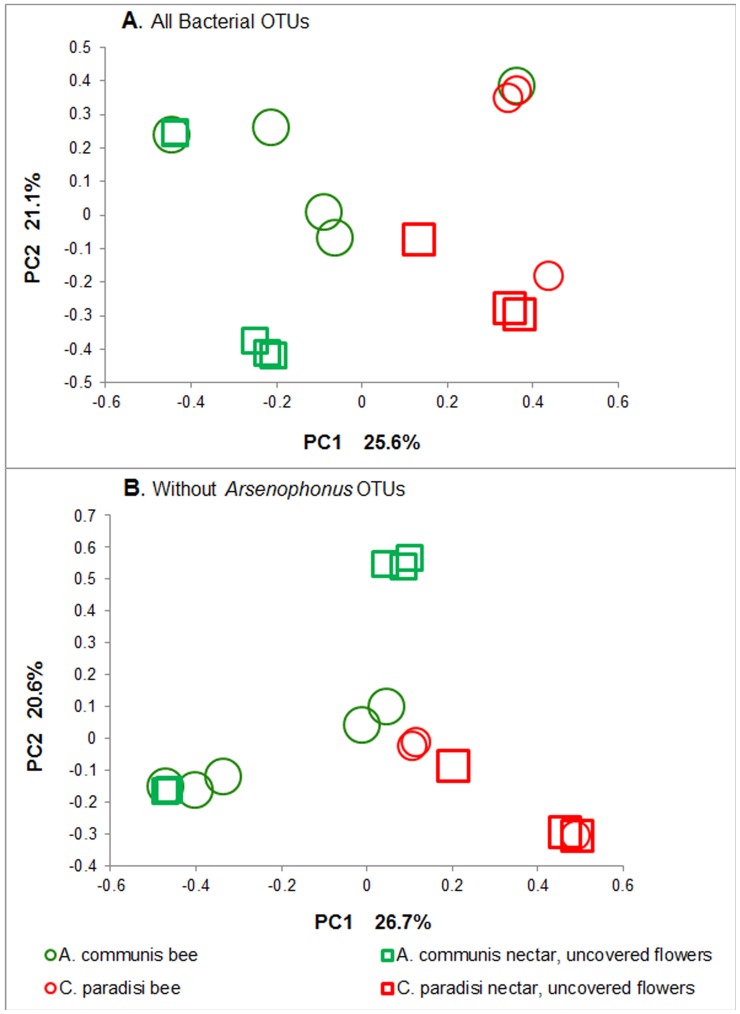
Nectar bacterial diversity clustering by plant species and sample source. (a) Analyses of all bacterial OTUs. (b) Analyses without *Arsenophonus* **OTUs.** (a) No significant differences were found in microbial communities among honeybees from the two different plants (F_1,6_ = 1.38 P = 0.191). However, each bee showed resembance only to the nectar of the plant it was captured on. (b) When data excluding the *Arsenophonus* OTUs were analyzed, significant differences were found in microbial communities among honeybees from the two different plants (F_1,6_ = 1.71 P = 0.013). Bacterial diversities of all samples were surveyed by 454-pyrosequencing of 16S rRNA genes. The first two principal coordinates (PC1 and PC2) from the principal coordinate analysis of weighted UniFrac are plotted for each sample. Each symbol represents a sample (circle, bee; square, nectar), colored by plant species (*A. communis*, green; *C. paradisi*, red). The variance explained by the PCs is indicated on the axes.

To compare the BCC of the two different plant species and of the bees captured on them, we excluded the covered flower data from the following analyses because they were derived from only one plant species. Principal component analysis (PCA) based on the relative abundance of the different bacterial phyla and classes attested to differences between the BCCs of the uncovered nectars of *A. communis* and *C. paradisi* flowers. The differences were found significant by AMOVA (F_1,6_ = 3.87; P = 0.012) (Fig. 4a). These two BCCs proved similar to those found on the honeybees captured from the respective plant species (supported by AMOVA: F_1,8_ = 1.61,P = 0.175 for *A. communis*; F_1,4_ = 1.93; P = 0.096 for *C. paradisi*).

No significant differences were found between the BCCs of honeybees from the two plant species (supported by AMOVA: F_1,6_ = 1.38 P = 0.191) (Fig. 4a). This similarity may be explained by the dominant presence of bacterial species from the genus *Arsenophonus* on bees captured on the flowers of the two plant species (Fig. 2). All the *Arsenophonus* sequences were removed from the main FASTA file, and were aligned in MOTHUR against the SILVA reference alignment [51]. To determine whether the removal of the *Arsenophonus* sequences affected the sampling depth of the community, we recalculated diversity (coverage) and the richness estimator (ACE) (Table S2). Sample diversity remained unchanged and sample richness was reduced less than 2%. The *Arsenophonus* sequences were divided into 1,669 different OTUs, of which 1,512 (90.2%) showed more than 98% resemblance to known species (for example: accession numbers DQ508172, DQ517447, DQ517448 and AY264669).

To support the hypothesis that the presence of *Arsenophonus* was the reason for the similarities of the BCCs in honeybees from the two plant species, we reanalyzed our data after excluding all OTUs representing the genus *Arsenophonus.* The results shown in Fig. 4b demonstrate that without *Arsenophonus,* there were significant differences between the BCCs in honeybees from the two plant species (supported by AMOVA: F_1,6_ = 1.71, P = 0.013) (Fig. 4b). The reanalysis yielded no significant differences between the nectar of uncovered flowers and bees captured from the respective plant species (as was found before exclusion of the *Arsenophonus* OTUs) (F_1,8_ = 1.67; P = 0.146 for *A. communis*; F_1,4_ = 0.91; P = 0.421 for *C. paradisi*).

## Discussion

### Bacterial Community in Nectar

In the current study we demonstrate that the bacterial community composition (BCC) in the nectar of two plant species was distinct (Fig. 4b). Similar results were obtained by Fridman et al. [10]. This interspecific variability in nectar’s BCC is reasonable as its chemical profiles, including minerals, sugars, secondary metabolites and pH, differ markedly among plant species (for example: 35,36), and therefore are likely to stimulate the growth and development of unique bacterial communities. Previous studies showed pronounced interspecific variability of phyllosphere BCCs [37]–[42], so different plant species may promote the colonization of different bacterial communities. Future studies should focus on the mechanisms that shape the unique BCCs in the nectar of diverse plant species.

*Acinetobacter* dominated the BCC in the floral nectar of *A. communis* and *C. paradise*, and were also found on honeybees which foraged on their flowers (Fig. 2). *Acinetobacter* was also the dominant genus in the BCC of floral nectar in our previous study [10] and in the studies of Alvarez-Perez et al. [11], [12], [43], suggesting that *Acinetobacter* species are abundant in bacterial communities of floral nectar.

### Bacterial Community on Bee Surfaces and Mouthparts

454-pyrosequencing of bacterial communities on bee surfaces revealed the presence of dominant OTUs belonging to the genus *Arsenophonus*; this caused the similarities between the BCCs in honeybees captured on the two different plant species (Fig. 4a). But when the sequences were analyzed without the *Arsenophonus* OTUs, the honeybees from the two plant species proved significantly different in their BCCs (Fig. 4b). The genus *Arsenophonus* is a widespread cluster of insect symbiotic bacteria [44], [45]. It has been described in honeybee intestines [25] but not on the body surfaces of bees or other insects. Nevertheless, it is possible that the source of these OTUs in the samples was the mandibles and the proboscis (tongue) that were pulled off and resulted in lysate traces of haemolymph and muscle cells which may have been released, together with the haemocoel. The presence of *Arsenophonus* in bee BCCs should be further studied.

Pyroseqencing analyses revealed that *Lactobacillus* spp. dominated the sample of bees captured from the *A. communis* plant (Fig 2). *Lactobacillus kunkeei* was also isolated and identified from the bees captured from both studied plant species (Table 1). This bacterial species was previously found in the stomach of honeybees [46], [47] and in flowers and honey [48]. However, *Lactobacillus kunkeei* was not isolated from the floral nectar of either of the studied plant. *Lactobacillus kunkeei* is an inhabitant of fructose-rich niches, so *C. paradisi* and *A. communis* nectar might not contain enough fructose for *L. kunkeei* growth.

### Do Honeybees Play the Role of Bacterial Vector between Floral Nectars?

The pioneering results of our study demonstrate the possible role of honeybees as bacterial vectors between flowers within a plant species.

Comparison of the BCCs by the 454-pyrosequencing method in the *C. paradisi* plant (bees vs. covered vs. uncovered flowers, Fig. 3) revealed that the BCC in the nectar of “unvisited” flowers was significantly different from that found in exposed nectar and on the bees captured on these flowers. AMOVA of pyrosequencing data without *Arsenophonus* found a better separation in the BCC on bees trapped on the flowers of the two plant species than did the analysis with *Arsenophonus* present. The BCCs from uncovered flower nectar and from bee surfaces and mouthparts on each plant species did not differ (Fig. 4). These results demonstrate that bees found foraging for nectar on a specific plant species carry a unique bacterial flora that resembles the bacterial flora in that specific plant nectar; hence, bees may act as bacterial vectors. The distinct BCCs we found on bees from the two plant species may also be explained by the fact that the plant species have different blossoming periods. Detzel and Wink [19] and Gruter et al. [20] noted that the individual bee tends to stick to one kind of flower over a certain period of time; however, it is possible that bees first visit *A. communis* (flowering around January), and begin visiting *C. paradisi* as it begins to blossom (around March), towards the end of the *A. communis* blossoming period.

Based on these results we suggest that honeybees have their own endogenous bacterial communities, as already demonstrated by others [24]–[27], [49]. Recently, McFrederick et al. [50] showed that honeybees and bumblebees have host-specific *Lactobacillus* associates. Nevertheless, apart from the fact that honeybees have their specific endogenous bacterial communities, the results of the current study suggest that they acquire bacteria from the environment they visit, such as the nectar of the flower of a specific plant species that they pollinate in a certain period. This behavior may cause the transfer of bacteria in and out of the nectar, thereby changing the BCC in the floral nectar. Furthermore, pollinator exposure gave rise to significant changes in the chemical profile of the nectar, such as sucrose–fructose balance and nectar pH [17], [52]. These results support our suggestion that the honeybee pollinator may possibly introduce bacteria into the nectar and thus may positively or negatively affect nectar quality and plant fitness.

In conclusion, we demonstrate that the BCCs in nectar are abundant and diverse (Table 1; Figs. 1 and 2). We also demonstrate that uncovered flowers show different bacterial populations than do covered flowers, indicating that the bacterial community in floral nectar is affected by insects which visit the flowers. The honeybee pollinator may introduce bacteria into, or acquire bacteria from, the nectar. Further research is needed to buttress this hypothesis and to explore the role of bacteria in the nectar in attracting or repelling honeybees or other nectar visitors.

## Supporting Information

Figure S1 Rarefaction curves indicating the observed number of operational taxonomic units (OTUs) at a genetic distance of 3% in different *Amygdalus communis* nectar samples (a) and bee samples (b). Capital letters B and N represent the sample origin: B- bee; N- floral nectar.
(TIF)

Figure S2 **Rarefaction curves** indicating the observed number of operational taxonomic units (OTUs) at a genetic distance of 3% in different *Citrus paradisi* samples. Capital letters B, C and U represent the sample origin: B- bee; C- nectar from covered flowers; U- nectar from uncovered flowers.
(TIF)

Table S1 **Sequences of primers and barcodes that were used for 454-pyrosequencing of the 16S rRNA gene.**
(DOC)

Table S2 **Coverage, chao1 and ACE richness estimator.**
(DOC)

Table S3 **OUT’s data.** OTU abundances and taxonomic classifications within each nectar or bee sample. Data was obtained using the 454 pyrosequencing.
(XLSX)
