# Supplementary figures and images for: Do Honeybees Shape the Bacterial Community Composition in Floral Nectar?
Source: PLoS One. 2013 Jul 3;8(7):e67556. doi: 10.1371/journal.pone.0067556 (PMC3701072; doi:10.1371/journal.pone.0067556)

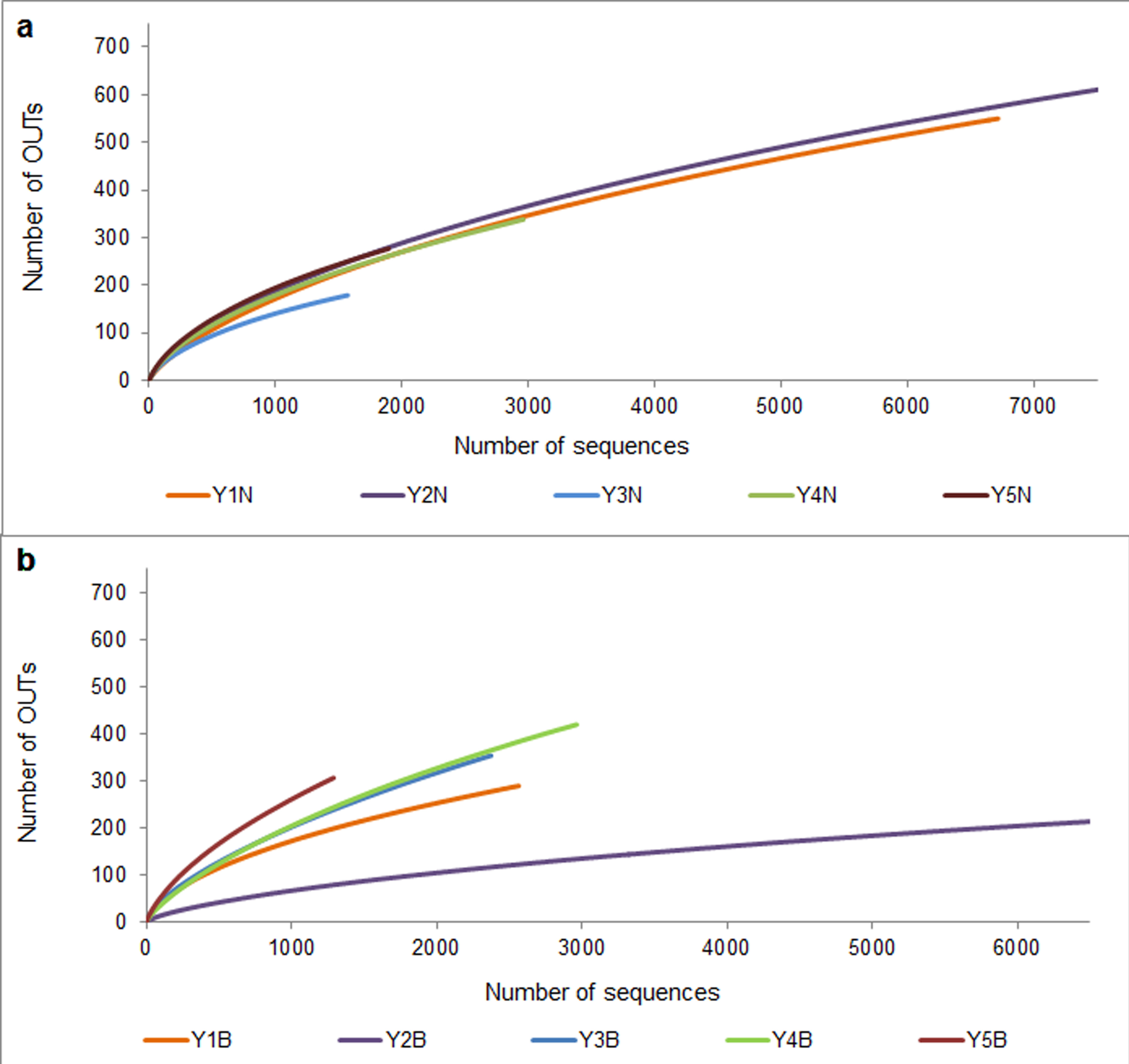

Supplement: Figure S1 — Rarefaction curves indicating the observed number of operational taxonomic units (OTUs) at a genetic distance of 3% in different Amygdalus communis nectar samples (a) and bee samples (b). Capital letters B and N represent the sample origin: B- bee; N- floral nectar. (TIF) [file pone.0067556.s001.tif]

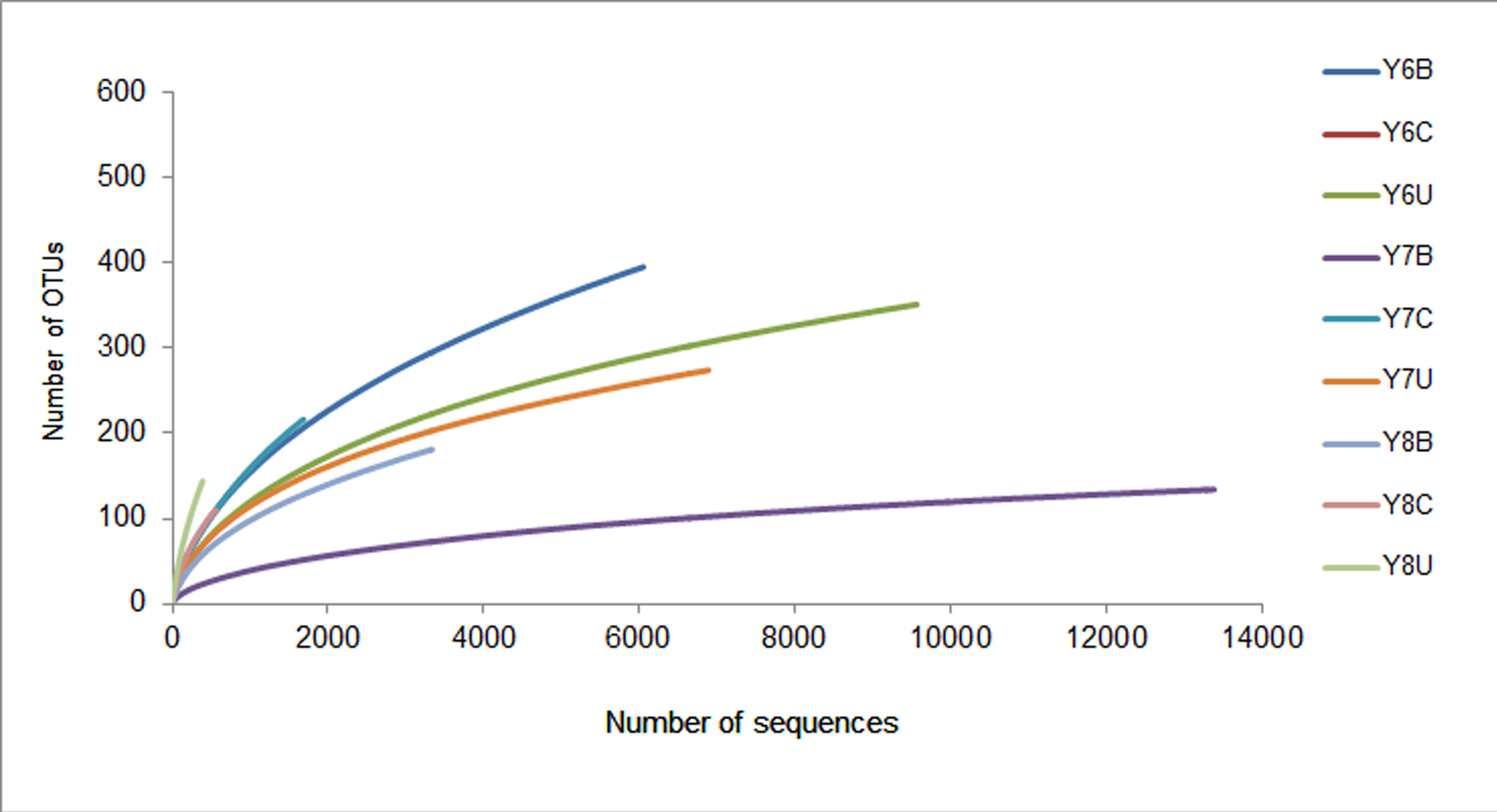

Supplement: Figure S2 — Rarefaction curves indicating the observed number of operational taxonomic units (OTUs) at a genetic distance of 3% in different Citrus paradisi samples. Capital letters B, C and U represent the sample origin: B- bee; C- nectar from covered flowers; U- nectar from uncovered flowers. (TIF) [file pone.0067556.s002.tif]
