# Supplementary material for: Do Honeybees Shape the Bacterial Community Composition in Floral Nectar?
Source: PLoS One. 2013 Jul 3;8(7):e67556. doi: 10.1371/journal.pone.0067556 (PMC3701072; doi:10.1371/journal.pone.0067556)
Supplement: Table S1 — Sequences of primers and barcodes that were used for 454-pyrosequencing of the 16S rRNA gene. (DOC) [file pone.0067556.s003.doc]

**Table S1. Sequences of primers and barcodes** that were used for 454-pyrosequencing of the 16S rRNA gene

| **Sample ID** | **Barcode Sequence** | **Linker Primer Sequence** |
| --- | --- | --- |
| Y1N | AAAAAAAA | GGCGVACGGGTGAGTAA |
| Y1B | AAAAAAAT | GGCGVACGGGTGAGTAA |
| Y2N | AAAAAAAG | GGCGVACGGGTGAGTAA |
| Y2B | AAAAAACC | GGCGVACGGGTGAGTAA |
| Y3N | AAAAAGTC | GGCGVACGGGTGAGTAA |
| Y3B | AAAAAGCG | GGCGVACGGGTGAGTAA |
| Y4N | AAAAAACT | GGCGVACGGGTGAGTAA |
| Y4B | AAAAAATA | GGCGVACGGGTGAGTAA |
| Y5N | AAAAAATC | GGCGVACGGGTGAGTAA |
| Y5B | AAAAAATG | GGCGVACGGGTGAGTAA |
| Y6C | AAAAAAGA | GGCGVACGGGTGAGTAA |
| Y6B | AAAAAAGC | GGCGVACGGGTGAGTAA |
| Y6U | AAAAAAGT | GGCGVACGGGTGAGTAA |
| Y7C | AAAAAAGG | GGCGVACGGGTGAGTAA |
| Y7B | AAAAACAA | GGCGVACGGGTGAGTAA |
| Y7U | AAAAACAC | GGCGVACGGGTGAGTAA |
| Y8C | AAAAAGCC | GGCGVACGGGTGAGTAA |
| Y8B | AAAAATAA | GGCGVACGGGTGAGTAA |
| Y8U | AAAAAGCT | GGCGVACGGGTGAGTAA |
