# Supplementary material for: Do Honeybees Shape the Bacterial Community Composition in Floral Nectar?
Source: PLoS One. 2013 Jul 3;8(7):e67556. doi: 10.1371/journal.pone.0067556 (PMC3701072; doi:10.1371/journal.pone.0067556)
Supplement: Table S2 — Coverage, chao1 and ACE richness estimator. (DOC) [file pone.0067556.s004.doc]

**Table S2. Coverage, chao1 and ACE richness estimator.**

| group | mean no. of seqs | coverage | coverage without *Arsenophonus* OTUs | ACE | ACE without *Arsenophonus* OTUs | chao1 |
| --- | --- | --- | --- | --- | --- | --- |
| *A. communis* nectar, uncovered flowers | 4138 | 94.23% | 93.08% | 1304.0 | 1170.03 | 757.1 |
| *A. communis*, bees | 3222 | 91.83% | 91.16% | 1303.9 | 1105.51 | 690.8 |
| *C. paradisi* nectar, uncovered flowers | 5612 | 90.90% | 90.24% | 1102.1 | 972.37 | 441.1 |
| *C. paradisi* nectar, covered flowers | 808 | 88.40% | - | 556.2 | - | 259.2 |
| *C. paradisi*, bees | 7588 | 97.92% | 97.11% | 874.6 | 757.14 | 430.7 |
